# TikTok and #OccupationalTherapy: Cross-sectional Study

**DOI:** 10.2196/45554

**Published:** 2023-05-19

**Authors:** Whitney Chasca, Samantha Nerada, Marco Zenone, Skye Barbic

**Affiliations:** 1 Department of Occupational Science and Occupational Therapy Faculty of Medicine The University of British Columbia Vancouver, BC Canada; 2 London School of Hygiene and Tropical Medicine London United Kingdom

**Keywords:** TikTok, occupational therapy, health professional, knowledge translation, social media, education, treatment, community, quality control, information, platform

## Abstract

**Background:**

Medical providers use the short-form video social media platform TikTok to share information related to their scope of practice and insights about their professions. Videos under the hashtag #occupationaltherapy on TikTok have over 100 million views, but there is no evidence investigating how occupational therapy information and knowledge are shared on the platform.

**Objective:**

The purpose of this cross-sectional study is to describe TikTok content with the hashtag #occupationaltherapy and investigate how occupational therapy is portrayed.

**Methods:**

We performed a content analysis on the top 500 TikTok videos under the hashtag #occupationaltherapy. We analyzed occupational therapy content themes (occupational therapy intervention, education, student training, universal design, and humor), practice settings (pediatrics, generalists, dementia, hand therapy, neurology, occupational therapy students, older adults, mental health, and unknown), and sentiments (positive, negative, and neutral).

**Results:**

The videos in our sample (n=500) received 175,862,994 views. The 2 most prevalent content areas were education (n=210) and occupational therapy interventions (n=146). The overall sentiment of the videos was positive (n=302). The most frequently observed practice settings in the videos were pediatrics (n=131) and generalists (n=129). Most videos did not state that it was occupational therapy (n=222) or misused the hashtag (n=131).

**Conclusions:**

TikTok has the potential for occupational therapists to share innovations, build communities of practice, and engage in collaborative efforts to share information about occupational therapists’ unique roles with diverse populations. Future research is needed to monitor the quality of information and debunk inaccuracies.

## Introduction

### Background

TikTok is a social media platform that was released in September 2017 [1] and allows users to produce and distribute short-form videos incorporating music, animation, and special effects on a variety of topics [2]. Increasing its user base faster than any other social media app since its inception 6 years ago [3], TikTok has become another effective platform to share and seek information worldwide [4]. Unlike most social media apps, TikTok is primarily controlled by algorithms, where content is personalized for each user based on previous and current viewing preferences [5,6]. The video time limit on TikTok increases engagement and interactive learning by disseminating information in a condensed and concise manner [7,8]. During the COVID-19 pandemic, organizations such as the World Health Organization (WHO) and other health care professionals were using this platform to convey important health information to the general public [9,10]. Numerous studies have analyzed the content of different health care professions on TikTok [9]. However, there are currently no studies that have examined the content related to occupational therapy on the platform to understand the information and knowledge being shared.

Occupational therapy enables individuals to attain their goals by providing them with solutions to overcome obstacles that can impede them from engaging in meaningful activities in their everyday lives [11,12]. The occupational therapy profession emerged during World War I when injured soldiers were provided with vocational training to help them return to a meaningful and self-sufficient life [13]. Since then, the profession has continued to develop and improve to meet the needs of diverse groups of individuals [11]. Despite occupational therapy originating over 100 years ago, there continue to be misperceptions surrounding the profession among other health care professionals and the general public [14-17]. This decreased awareness may result in a lack of understanding and underappreciation for the profession’s scope of practice and the benefits it brings.

### Aim

As of April 2022, the hashtag #occupationaltherapy had 175,862,994 views on TikTok. Given this statistic and the ability of TikTok to reach large audiences, this study can further the profession’s understanding of how social media apps can raise awareness of occupational therapy. In response to the gap in the research literature, the purpose of this study is to understand the sentiment and content of videos on TikTok with the hashtag #occupationaltherapy to determine what information is being shared and how occupational therapy is being portrayed.

## Methods

### Data Collection

**Figure 1:**
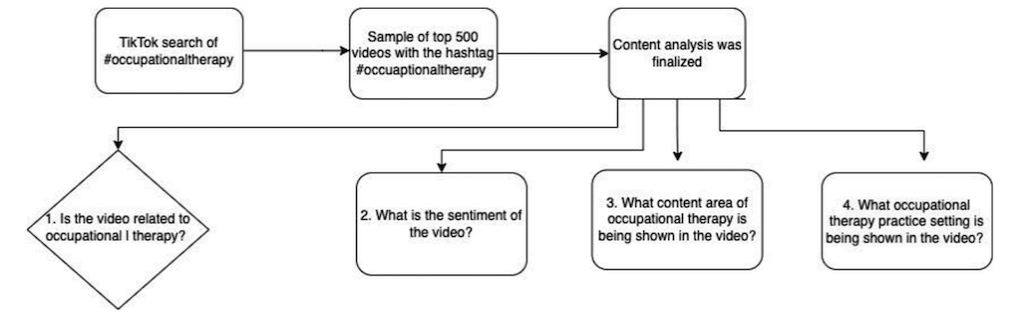
Flow diagram representing the research process.

As shown in Figure 1, this cross-sectional study analyzed and coded the top 500 English-language TikTok videos under the hashtag #occupationaltherapy. From April 13-14, 2022, one team member (MZ) scraped the URLs of the top 500 videos with the hashtag #occupationaltherapy using the program DataMiner (Software Innovation Lab LLC). MZ then performed a second scrape to pull the metadata of the videos, including the views, likes, and all other information available on the platform. Videos were manually downloaded and assigned a unique number that corresponded with their metadata in a Microsoft Excel worksheet. The sample included the hashtag search results a user would see if they demonstrated an interest in occupational therapy by searching occupational therapy-related hashtags. The inclusion criteria for the videos were those in English and with the hashtag #occupationaltherapy. All videos selling products and non–English-speaking videos were excluded (n=40). After implementing the exclusion criteria, the final sample resulted in 460 videos, which was deemed an appropriate sample size based on previously conducted studies of similar nature [18-20]. The collection and distribution of the data were in accordance with TikTok’s terms and conditions and did not require a secure platform to store the data as the information is accessible to the general public. The data were categorized and analyzed using Microsoft Excel (Microsoft Corporation) and then inputted into Microsoft Word to visualize and analyze the results.

### Ethical Considerations

This study did not require research ethics approval because all TikTok data, including photos, was posted publicly with no expectation of privacy.

### Study Design

This study was completed using content analysis with a grounded theory approach [21]. For our content analysis, team members SN and WC analyzed the videos for information related to the occupational therapy scope of practice. We immersed ourselves in the data by viewing the videos repeatedly to create categories that were most representative of the content of occupational therapy being represented on TikTok. We continued with this approach until no new categories were created and saturation was reached.

#### Data Analysis

Authors SN and WC collectively coded 100 videos and generated categories encompassing the occupational therapy scope of practice represented on TikTok. Through this process, a content analysis framework was created. The coding framework was finalized in May 2022 after review and feedback from MZ and SB, who then made minor refinements. All authors agreed on the final coding frame, which collected information on the following video characteristics being illustrated by the TikTok videos: the overall sentiment, content area, and the practice setting. Researchers sought to understand the overall sentiment of the videos and whether the data representing the occupational therapy profession had a positive, negative or neutral sentiment. The content areas were selected based on the common themes that emerged in the videos where occupational therapy was being represented which can be seen in Table 1. The content areas were confirmed with the guidance of the Occupational Therapy Act [22], which provided a detailed description of the scope of occupational therapy. Practice settings for occupational therapy were selected based on the areas where occupational therapists were most frequently seen working, as outlined by the Canadian Association of Occupational Therapists [23]. Details of the overall video characteristics selected are described in Textbox 1. Additionally, researchers were interested in understanding whether the TikTok videos that incorporated the hashtag #occupationaltherapy were in fact related to occupational therapy, or if the hashtag was being misused.

The remaining 400 videos were equally divided, independently coded, and analyzed by SN and WC. For the purpose of this study, we discontinued further coding when videos were unrelated to occupational therapy. SN and WC randomly selected 100 videos from their counterparts to audit and assess the consistency of code application decisions. Following the audit, the coders agreed on 89% of coding decisions, showing high agreement [24]. Disagreements were resolved through internal discussion between SN and WC or through consultation with SB. The coding was completed in August 2022.

**Table 1:** What occupational therapy content is being represented in the videos?

Content area			Description
**Occupational therapy intervention**			Different treatments that occupational therapists can provide to individuals
	Fine motor skills	Activities that target the smaller muscles of the hands, wrists, and fingers	
	Gross motor skills	Activities that target the larger muscle groups	
	Assistive technology	Devices or pieces of equipment that improve function for individuals with disabilities	
	Hand therapy	Interventions that target the hand and arm specifically	
**Education**			Video explained, taught, or gave advice related to occupational therapy, or advocated for the profession
	Pediatric tips and education	Video provided education on the benefits of occupational therapy for children by providing viewers with education, advice, and activities that are related to occupational therapy and childhood diagnoses	
	General tips and education	Health information, education, and tips related to the occupational therapy scope of practice for individuals of different ages without a clear diagnosis	
	Dementia tips and education	Different activities, tips, and education for individuals directly or indirectly affected by dementia	
Student training			Tips and strategies for students studying occupational therapy to help promote success
Universal design			Adapting or designing environments or products to make them more inclusive for all individuals
Humor			Both positive and negative illustrations of occupational therapy through the use of comical anecdotes
Unknown			It was unclear what content area was being illustrated

**What occupational therapy information is being represented on TikTok?**

**Positive or negative sentiment**

- Was the sentiment of the video positive, negative, or neutral?

**Occupational therapy content area**

- What topics or activities related to occupational therapy were being shown or taught?

**Occupational therapy practice settings**

- What occupational therapy practice setting was being represented?

## Results

### Overview

**Figure 2:**
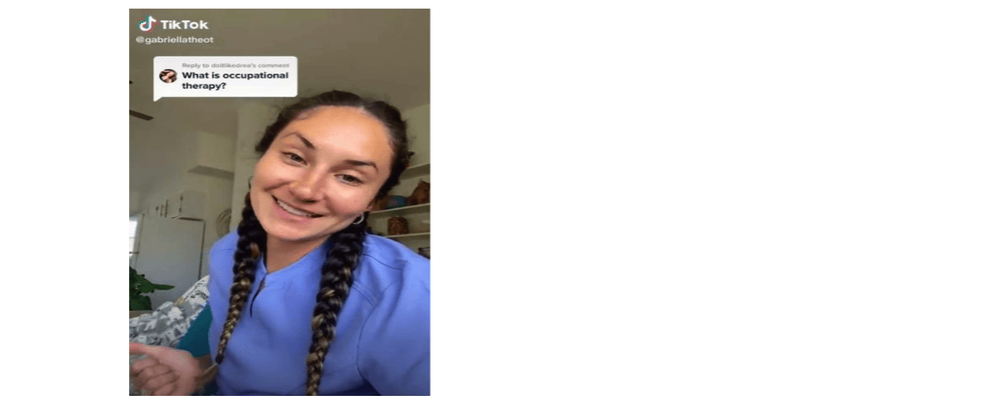
Video directly states occupational therapy through verbal and written statements.

**Figure 3:**
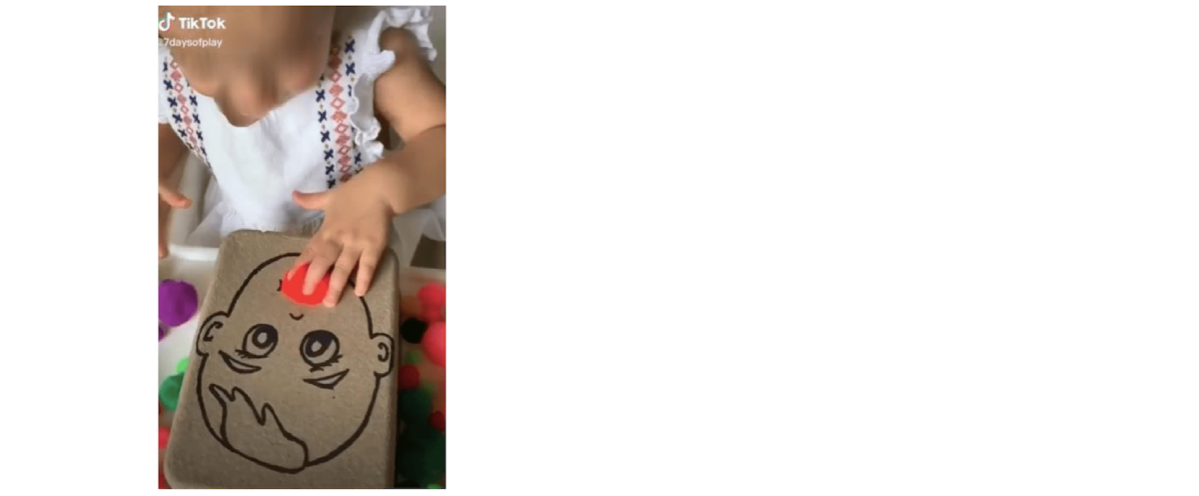
Video shows a fine motor activity that occupational therapists often use for treatment and interventions but does not clearly indicate relevance to occupational therapy.

**Figure 4:**
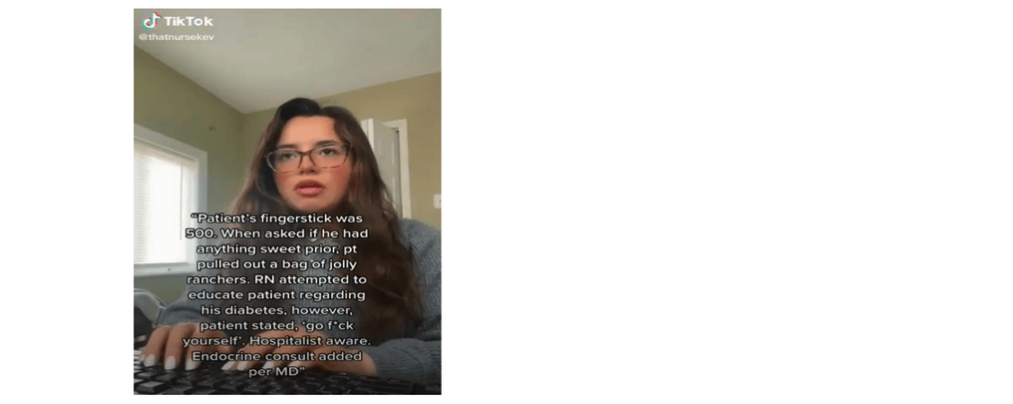
Video shows a registered nurse (RN) who used the hashtag #occupationaltherapy despite it being unrelated to occupational therapy.

Of the 460 TikTok videos that were analyzed, 23% (n=107) were directly disclosed as being related to the occupational therapy profession through verbal or written statements (see Figure 2). Despite being within the scope of occupational therapy, 48% (n=222) of the videos did not clearly indicate that the content was related to the profession (see Figure 3). The remaining 29% (n=131) of videos were not related to occupational therapy and incorrectly used the hashtag #occupationaltherapy (see Figure 4). Videos in this category included creators sharing personal stories unrelated to occupational therapy or those of other health care professionals.

### The Overall Sentiment

The main sentiment expressed in the TikTok videos with the hashtag #occupationaltherapy was positive (302/460, 66%). Only 4% (17/460) of the videos had a negative sentiment, and even fewer were considered neutral (10/460, 2%). Since the remaining videos were not coded further after being categorized as being unrelated to occupational therapy, 28% (131/460) of the videos in our sample did not have a sentiment attached to them because they were unrelated to occupational therapy. The creators of the videos with a negative sentiment either challenged the credibility of an occupational therapist (n=5), had a misperception of the profession (n=5), expressed patient dissatisfaction with occupational therapy services (n=5), or described the disadvantages of being an occupational therapist (n=2). Textbox 2 provides examples of the types of TikTok videos that were considered to have positive, neutral, and negative sentiments.

**Examples of videos with positive, neutral, and negative sentiments.**

**Positive, neutral, and negative sentiments from the videos**

- Positive
  Video asking “What is occupational therapy?” and explaining the benefits of occupational therapy.
  Video showing a child having a positive experience with occupational therapy.
  Video demonstrating a mother of a child seeing benefits from occupational therapy treatment.
  Video showing a tactile guide to help individuals with vision impairments type on a keyboard more easily.
  Video showing a child completing a fine motor activity.
  Video demonstrating how to use a sock aid.
  Video providing encouragement to occupational therapy students who feel stressed and unmotivated.
  Video educating caregivers to teach someone with dementia to use a walking aid.
- Neutral
  Video showing reels of photos related to occupational therapy school.
  Video stating “Conversations I have with parents as a pediatric occupational therapist,” followed by a variety of examples.
  Video stating, “If this video could make it to occupational therapy, TikTok…” related to parents with back problems from having children.
  Video showing an assistive device but not explaining its purpose or benefits.
- Negative
  Video questioning “Is therapy harming my child?”
  Video stating, “Why is my therapist making me do this?”
  Video discrediting the occupational therapy profession by saying, “Oh, you’re studying occupational therapy; could you not get into physiotherapy?”
  Video demonstrating dissatisfaction with occupational therapy services.
  Video demonstrating a misunderstanding of the occupational therapy profession by a doctor asking an occupational therapist to do nursing responsibilities.

**Occupational Therapy Content**

The 2 main occupational therapy content areas that appeared in the TikTok videos were education and occupational therapy interventions. Education was the most prominent content area found in the analysis and was seen in 210 videos. Videos classified as educational included creators providing occupational therapy tips and education for different populations. The 3 subcategory topics that emerged from education provided information on the following areas: pediatrics (97/460, 21%), general occupational therapy information (89/460, 19%), and dementia (24/460, 5%). Occupational therapy interventions appeared in 146 videos that demonstrated the different treatments occupational therapists provide. These interventions include assistive technology (55/460, 12%), hand therapy (26/460, 6%), fine motor skills (24/460, 5%), and gross motor skills (15/460, 3%). Note that several other interventions related to occupational therapy were seen but were eliminated from Table 1 if they appeared in less than 2% of the videos. These interventions included content related to hand strengthening (n=8), activities of daily living and instrumental activities of daily living (n=7), handwriting (n=5), stroke (n=5), range of motion interventions (n=4), grading and chaining (n=4), sensory modulation (n=4), transfer training (n=2), stretching (n=2), desensitization (n=2), taping for shoulders (n=2), dysphagia (n=1), therapy for tone (n=1), electrical stimulation (n=1), and wheelchair safety and training (n=1). Some videos were coded into multiple categories if researchers thought the video encompassed more than one educational area or intervention.

The content areas that were seen infrequently appeared in less than 25 videos are humor, student training, universal design, and unknown (Table 1). Humorous content was seen in 24 videos, where creators comically described aspects of occupational therapy. These findings included content related to general humorous responses from clients (n=9), jokes about occupational therapy as a profession (n=3), comical interactions with clients (n=2), health care professionals making jokes specifically about occupational therapists (n=2), jokes about other health care professionals (n=1), and people having a misperception of the profession (n=1). The additional videos were categorized as being unspecific humor related to occupational therapy (n=6). Notably, 29% (7/24) of the humorous videos had a negative sentiment. Student training appeared in 21 videos in this sample, where creators were seen providing tips to occupational therapy students in the following areas: note-taking (n=4), providing positive encouragement (n=4), passing exams (n=1), and interviewing (n=1). Videos that demonstrated what a day in the life of an occupational therapy student looks like were also included in this category (n=3) along with videos that fit the category of content related to prospective occupational therapy students (n=8). Universal design was one of the least common content areas seen in our study (n=4), which included videos illustrating home modifications, accessible bathrooms, and playgrounds. Videos were categorized as unknown (n=8) if the creator did not provide a clear explanation of the content being illustrated.

### Occupational Therapy Practice Settings

The occupational therapy practice settings that were most commonly seen in the TikTok videos were pediatrics (n=131), generalists (n=129), dementia (n=25), and hand therapy (n=20). The descriptions of these practice settings are defined by the Canadian Association of Occupational Therapists and are located in Table 2. The other practice settings that appeared in the videos included content related to neurology (n=11), occupational therapy students (n=7), older adults (n=2), mental health (n=2), or were unknown (n=2) and can also be seen in Table 2.

**Table 2:** What occupational therapy practice setting is being represented?

Practice setting	Description	Videos, n
Pediatrics	Pediatric occupational therapy “assesses areas of fine and gross motor skills, cognitive skills, social development, mental health, and establishing self-care routines. Uses a holistic and family-centered approach to implement treatment plans that are based on the child’s interests and needs” [23].	131
Generalist	Occupational therapists in a generalist role “practice in a wide variety of contexts with competency and skills that are used across the life span (eg, children, adolescents, adults)” [23].	129
Dementia	Occupational therapists working in dementia care are professionals that “assess… difficulties with day-to-day activities and work with the client and family to implement strategies to assist with memory, managing challenging behaviors, falls prevention, mealtime activities, medication management, and communication” [23].	25
Hand therapy	A practice area that “treat[s] conditions that impact the functional use of the arm and hand” [23].	20
Neurology	Occupational therapists working in neurology settings help to “facilitate individuals living with neurological conditions [for example] Spinal Cord Injury (SCI), Amyotrophic Lateral Sclerosis (ALS), Stroke, and Parkinson Diseases, to maximize participation in meaningful daily activities” [23].	11
Occupational therapy students	Occupational therapy students or therapists who provide education, tips, and strategies for students studying occupational therapy.	7
Older adults	Occupational therapists that work with older adults (65 years and older) typically practice in “a variety of settings such as hospitals, community, long-term [care] facilities, and palliative care.” They provide “rehabilitation techniques and mental health support to address barriers related to aging and to assist in daily tasks such as dressing, eating, and bathing” [23].	2
Mental health	The practice area of mental health involves occupational therapists supporting “recovery by providing strategies to help clients cope with everyday activities and stressors, [and] assesses skills, interests, values, and strengths to help clients maintain, modify, or participate in meaningful occupations” [23].	2
Unknown	This category was included to capture the TikTok videos with the hashtag #occupationaltherapy where the practice setting was not clearly disclosed or identifiable.	2

## Discussion

### Principal Findings

The findings of our study suggest important implications for the clinical practice of occupational therapists. Similar to other social media platforms, TikTok can be an effective way to deliver public health information to diverse populations [25-28]. Many previous studies have analyzed TikTok content as it relates to other health care areas such as dermatology, diabetes, attention-deficit/hyperactivity disorder, and cancer [18,20,29,30]. However, this is the first study to explore what occupational therapy information and knowledge are being shared on TikTok. Although these social media platforms have the potential to disseminate important information worldwide, viewers should exercise caution when using these apps as the quality and reliability of the information are often unknown [28,31]. Previous studies that analyzed various aspects of public health information on TikTok have found a high percentage of videos spreading misinformation [20,29,32-35]. TikTok prohibits harmful medical misinformation [36] and allows users to report misinformation, but it receives criticism for a large amount of misinformation still spreading despite its policies [37].

Our study did not audit for misinformation related to occupational therapy but rather examined the misuse of the hashtag #occupationaltherapy by TikTok creators. The findings revealed that many other health care professionals inaccurately used the hashtag #occupationaltherapy. Having creators misuse the hashtag #occupationaltherapy may misrepresent the occupational therapy scope of practice and contribute to an inaccurate or misinformed representation of the profession [38,39]. Additionally, while the study found that many of the analyzed TikTok videos were within the occupational therapy scope of practice, nearly half of the videos did not explicitly indicate that the video content was related to occupational therapy, making it unclear to those unfamiliar with the profession. Only a small percentage of videos (107/460, 23%) directly disclosed the content as being related to occupational therapy. By not clearly identifying the content to be related to occupational therapy, the opportunity to showcase the breadth of the profession may be missed, which may discredit the value of occupational therapy.

Most of the videos examined in our study provided education to the viewers on topics or interventions related to occupational therapy. Similar to our findings, previous studies that examined public health information on TikTok found that many of the videos were also intended to provide education to the viewers [8,25,40]. It is important that creators declare that the information they are sharing on the platform is related to occupational therapy to increase awareness surrounding the profession and scope of practice. Additionally, pediatrics and generalists were the most identified practice settings in our sample. However, there are a variety of additional settings where occupational therapists work [23]. If used properly, TikTok could be an effective tool for occupational therapists to educate people about the many benefits occupational therapy has to offer and the different settings in which they can work.

### Limitations

There are a variety of limitations to our study. Our sample was limited to English-language videos only. However, occupational therapy is a worldwide profession and is not restricted only to English-speaking countries. It is also important to note that our coding collection may have been limited by subjective interpretations. The video sample used in this study was determined by TikTok’s listing of the first 500 videos with the hashtag #occupationaltherapy in April 2022. However, it is important to note that the views and engagement of these videos are constantly evolving, meaning that the findings of this study would likely vary if they were replicated at a different time. It can also be assumed that videos that were not included in the top 500 may have contained valuable information related to occupational therapy that was not accounted for in our reported findings. Lastly, only the hashtag #occupationaltherapy was scrutinized, which limited our data collection to 1 hashtag with a specific phrase.

### Clinical Implications

The potential reach that social media apps have is profound, especially with TikTok being one of the fastest-growing social media apps [3]. As noted in Textbox 3, when used correctly, occupational therapists can harness the power of TikTok to further create communities of practice and use it as an opportunity to connect and raise awareness of the profession with people worldwide. There is a clear opportunity for the advancement of the profession by using TikTok to share innovations and best practices, build communities of practice, and engage in collaborative efforts to share information about their role with diverse populations. This is the first known study to capture the portrayal of occupational therapy and analyze the content being shared on TikTok. There is potential for future research to examine the accuracy of the content of occupational therapy information on TikTok to determine if misinformation is being disseminated. There is a strong urgency for regulating bodies to provide content creation guidelines to occupational therapists to enable them to different social media apps to share information internationally. Researchers urge the profession to pay close attention to the quality control and accuracy of occupational therapy-related content on social media platforms.

**Key recommendations for the occupational therapy profession.**

**Key recommendations**

- Content creation guidelines from regulating bodies to train occupational therapists to use social media platforms to share occupational therapy information
- Regulating bodies create workshops that educate occupational therapists to verify the credibility and accuracy of the videos being shared on social media apps and increase their awareness of the potential consequences of sharing misinformation about the profession
- To increase engagement with occupational therapy videos, content creators should keep the videos short (under 1 minute) and use the hashtag #occupationaltherapy
- To clearly represent the scope of occupational therapy and to increase awareness surrounding the profession, content creators should clearly indicate that the content is related to occupational therapy, either through verbal or written statements

## Abbreviations

CAOT: Canadian Association of Occupational Therapists
